# 灰区淋巴瘤5例报告并文献复习

**DOI:** 10.3760/cma.j.issn.0253-2727.2023.03.011

**Published:** 2023-03

**Authors:** 鹤松 邹, 洪菊 张, 慧敏 刘, 文阳 黄, 薇 刘, 瑞 吕, 婷玉 王, 伟薇 隋, 明伟 傅, 齐 王, 录贵 邱, 德慧 邹

**Affiliations:** 中国医学科学院北京协和医学院血液病医院（中国医学科学院血液学研究所），实验血液学国家重点实验室，国家血液系统疾病临床医学研究中心，细胞生态海河实验室，天津 300020 Institute of Hematology & Blood Diseases Hospital, Chinese Academy of Medical Sciences & Peking Union Medical College, State Key Laboratory of Experimental Hematology, National Clinical Research Center for Blood Diseases, Haihe Laboratory of Cell Ecosystem, Tianjin 300020, China

**Keywords:** 淋巴瘤，B细胞, 纵隔, 药物疗法，联合, Lymphoma, B-cell, Mediastinal, Drug therapy, combination

## Abstract

**目的:**

探索灰区淋巴瘤（GZL）的临床及病理特征、治疗及预后。

**方法:**

回顾性分析2013年7月2日至2021年2月10日就诊于中国医学科学院血液病医院的5例灰区淋巴瘤患者的临床表现、病理学特征、治疗及转归情况。

**结果:**

男1例，女4例，中位年龄28（16～51）岁。4例患者纵隔（胸腺）受累，其中2例伴上腔静脉阻塞综合征，3例伴结外受累。Ann Arbor分期局限期1例，进展期4例。3例患者的病理形态与经典型霍奇金淋巴瘤（cHL）类似，可见霍奇金样细胞散在分布，免疫表型为CD20强表达，CD30表达而CD15不表达；另2例患者形态兼具cHL和弥漫大B细胞淋巴瘤（DLBCL）特征，部分区域形似霍奇金细胞，部分区域则形似免疫母细胞，免疫表型CD20表达强弱不等，CD30和CD15强表达。2例患者采用cHL样方案诱导治疗，仅获得部分缓解；予DLBCL样增强免疫化疗方案挽救治疗后均获得完全缓解（CR）。3例患者给予增强的DLBCL样免疫化疗方案诱导治疗，2例患者有效，其中1例获得CR。4例未获得CR的患者接受二线或三线挽救治疗后均获得CR，其中3例给予自体造血干细胞移植（ASCT）巩固治疗。1例患者失访，1例患者在诊断后35.9个月时因疾病进展死亡，其余3例患者均维持持续缓解状态。

**结论:**

GZL罕见，多发生于年轻患者，纵隔受累常见，诊断依赖病理形态和免疫表型。初诊GZL可能对DLBCL样强化免疫方案更敏感，复发难治患者可考虑非交叉耐药的联合化疗或联合新药。

## Abstract (en)

**Objective:**

To investigate the clinical and pathological features, treatment, and prognosis of gray zone lymphoma（GZL）.

**Methods:**

From July 2, 2013, to February 10, 2021, the clinical and pathological features, treatment, and outcomes of five patients with GZL at the Blood Diseases Hospital, Chinese Academy of Medical Sciences were studied retrospectively.

**Results:**

There were one male and 4 females, with a median age of 28（16−51）years at diagnosis. Four patients had mediastinal（thymic）involvement, two of which had superior vena cava obstruction syndrome, and 3 patients had extra-nodal involvement. There was one case with a limited Ann Arbor stage and 4 cases with a progressive stage. Three patients had cHL-like pathomorphology with scattered Hodgkin-like cells, strongly positive for CD20, positive for CD30, and CD15 was negative; the other two patients had both cHL and DLBCL morphology, with some areas resembling Hodgkin cells and some areas resembling immunoblasts, strongly positive for CD30, and CD15 but negative CD20. Two patients were treated with cHL-like regimens for induction and achieved only partial remission; after salvage therapy with enhanced DLBCL-like regimens, all achieved complete remission（CR）. Three patients were treated with enhanced DLBCL-like immunochemotherapy regimens for induction, and two patients were effective, one of whom achieved CR. Four patients who did not achieve CR were given second or third-line salvage therapy, and all of them recovered. One patient lost parity, one died of disease progression at 35.9 months after diagnosis, and the remaining three maintained sustained remission.

**Conclusion:**

GZL is uncommon, usually affects younger patients, is mediastinal and is diagnosed using path morphology and immunophenotype. Patients with newly diagnosed GZL appear to be more sensitive to DLBCL-like immunochemotherapy regimens; relapsed or refractory patients were tended with non-cross-resistant combination chemotherapy or with new drugs.

既往灰区淋巴瘤（GZL）包含两类淋巴瘤，分别是介于弥漫大B细胞淋巴瘤（DLBCL）和经典型霍奇金淋巴瘤（cHL）之间的不能分类的B细胞淋巴瘤及介于DLBCL和伯基特淋巴瘤（BL）之间不能分类的B细胞淋巴瘤。但在2016年WHO淋巴组织肿瘤新分类中，后者被纳入高级别B细胞淋巴瘤的范畴，而GZL特指介于DLBCL和cHL之间的不能分类的B细胞淋巴瘤[1]，其临床表现、形态学、免疫表型特征及分子遗传学特征介于DLBCL或原发纵隔大B细胞淋巴瘤（PMBCL）和cHL之间。但根据最新2022年WHO分型，GZL进一步被定义为需存在纵隔受累，即纵隔灰区淋巴瘤（MGZL）[2]。考虑到GZL临床罕见，诊断及治疗具有一定困难，为了提高对该疾病的认识，本文回顾性分析了我科收治的5例GZL患者，并进行文献复习。

## 病例与方法

1. 病例：2013年7月2日至2021年2月10日就诊于中国医学科学院血液病医院的5例GZL患者，收集其临床表现、影像学资料、病理学特征及治疗转归情况等。

2. 病理相关检测：淋巴结石蜡组织切片由两名经验丰富的病理科医师会诊，结合病理形态和免疫组织化学染色（IHC），依据WHO 2017修订版淋巴造血系统肿瘤分类进行诊断复核。IHC选用的抗体包括CD20、CD79a、CD3、CD5、Ki-67、CD15（中山市澳泉医疗科技有限公司产品），PD1、CD30、PAX-5（上海杰浩生物技术有限公司产品）、MUM1（北京中杉金桥生物技术有限公司产品）等。染色体荧光原位杂交（FISH）使用CytoCell双色标记TP53探针（英国Cytocell公司产品），VYSIS双色分离MYC、BCL2和BCL6探针（美国Abbott公司产品）检测相应基因异常。通过Illumina NextSeq 550测序平台对与淋巴瘤密切相关的125个基因蛋白编码区域的点突变和短片段插入/缺失突变进行高通量测序，平均测序深度为2 000×。

3. 疗效评价标准：参照2014版修订的Lugano淋巴瘤疗效标准评判疗效[3]，分为完全缓解（CR）、部分缓解（PR）、疾病稳定（SD）、疾病进展（PD）。

4. 随访：随访截止时间为2021年12月30日，中位随访36.9（10.7～102.4）个月。采用查阅患者住院病历和电话联系的方式进行随访。无进展生存（PFS）时间定义为自诊断至发生PD或复发、因任何原因死亡或末次随访的时间。总生存（OS）时间定义为自诊断至因任何原因死亡或末次随访的时间。

## 结果

1. 临床特征：5例患者中位年龄28（16～51）岁，男1例，女4例。所有患者均因浅表淋巴结肿大就诊，2例患者伴胸闷、喘憋（CT提示胸腔积液，伴上腔静脉综合征），3例伴发热。4例患者纵隔（胸腺）受累，3例患者伴结外受累（1例肋骨、肺，1例小肠，1例肋骨）。Ann Arbor分期局限期1例，进展期4例。可获得IPI评分的患者中2例为中低危，2例为中高危或高危组（表1）。

**表1:** 5例灰区淋巴瘤患者的临床及病理学特征

例号	性别	年龄（岁）	临床分期	IPI评分	受累部位			病理形态		免疫表型					一线方案	疗效	二线及以上治疗及治疗反应	转归	PFS（月）	OS（月）
					纵隔	外周淋巴结^a^	结外部位	类型^b^	生长方式^c^	CD20	PAX5	CD79a	CD30	CD15						
1	女	28	ⅢA	2	是	A、C、I、SM	–	1	D	+	+	ND	+	–	ABVD×4	PR	R-CVAD、EPOCH，CR	失访	–	–
2	男	37	ⅣB	4	是	A、C、SC、AC	肺、肋骨	1	D	+	+	ND	+	–	R-CHOPE×7	PR	R-DHAP×2，PD；BEACOPP×5、ASCT，CR	存活	6.8	102.4
3	女	16	ⅣA	2	是	A、C	肋骨	2	N	+	弱+	+	+	+	R-EPOCH×6序贯ASCT	CR	–	存活	88.8	88.8
4	女	51	ⅣA	NA	否	C、AC	肠道	1	N	+	弱+	ND	+	NA	BEACOPP×6	PR	R-EPOCH×4、ASCT，CR	复发死亡	35.9	36.9
5	女	25	ⅡB	3	是	A、C、SC、AC	–	2	D	+	+	弱+	+	+	R-EPOCH×2	PD	GDP+BV×3，PD；Benda+PD1×2、ASCT，CR	存活	2.5	10.7

**注** ^a^外周淋巴结：A：腋窝，C：颈部，I：腹股沟，SM：颌下，SC：锁骨上，AC：腹腔；^b^病理形态类型1：经典型霍奇金淋巴瘤（cHL）形态（小淋巴细胞增生背景上可见异型细胞散在分布，形态类似里德-斯特恩伯格细胞），类型2：cHL和弥漫大B细胞淋巴瘤（DLBCL）形态（兼具cHL和DLBCL的特征，异型细胞广泛或结节状分布，核分裂象易见，背景细胞丰富）；^c^生长方式：D：弥漫性，N：结节状；NA：未知；ND：未检测。ABVD：阿霉素+博来霉素+长春花碱+达卡巴嗪；R：利妥昔单抗；CHOPE：环磷酰胺+阿霉素+长春新碱+泼尼松+依托泊苷；EPOCH：依托泊苷+环磷酰胺+阿霉素+长春新碱+泼尼松；BEACOPP：博来霉素+依托泊苷+阿霉素+环磷酰胺+长春新碱+丙卡巴肼+泼尼松；CVAD：环磷酰胺+阿霉素+长春新碱+地塞米松；DHAP：地塞米松+大剂量阿糖胞苷+顺铂；GDP：吉西他滨+地塞米松+顺铂；BV：维布妥昔单抗；Benda：苯达莫司汀；PD1：PD1单克隆抗体；ASCT：自体造血干细胞移植；PR：部分缓解；CR：完全缓解；PD：疾病进展；PFS：无进展生存时间；OS：总生存时间

2. 病理形态及免疫表型：3例患者形态类似cHL，在小淋巴细胞、组织细胞等背景上可见霍奇金样肿瘤细胞散在或疏松的簇状分布，该类细胞多数胞体大，胞质丰富，胞核椭圆或略不规则，免疫表型为CD20强表达，CD30表达而CD15不表达。2例患者形态兼具cHL和DLBCL特征，部分区域形似霍奇金细胞，部分区域则形似免疫母细胞，可见双核及多核肿瘤细胞，伴有不同程度纤维化和坏死灶，免疫表型CD20表达强弱不等，CD30和CD15强表达（表1、图1）。

**图1:**
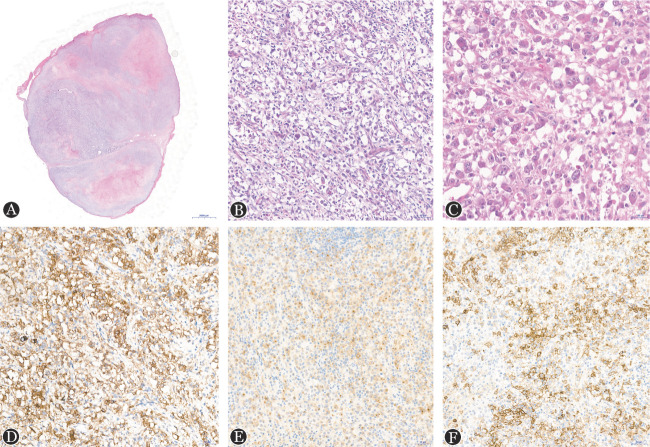
例5颈部淋巴结病理组织学形态及免疫表型 **A** 颈部淋巴结HE染色，低倍镜下可见纤维组织增生和灶性坏死（×7）；**B** 肿瘤细胞呈片状增生（HE染色，×200）；**C** 肿瘤细胞形态多样，部分形态似免疫母细胞，部分形态似霍奇金细胞，可见双核及多核肿瘤细胞（HE染色，×400）；**D** 肿瘤细胞CD30阳性（×200）；**E** 肿瘤细胞CD15阳性（×200）；**F** 肿瘤细胞CD20异质性表达（×200）

3. 染色体核型及分子遗传学：5例患者染色体核型分析均正常。2例患者淋巴结组织石蜡切片行FISH检测TP53、MYC、BCL2及BCL6基因均未见异常。1例患者淋巴结组织石蜡切片行二代测序检测可见B2M和TNFAIP3热点突变，突变频率分别为8.10％和11.20％。

4. 治疗及转归：所有患者一线均接受联合化疗或免疫化疗。2例患者采用cHL样方案（BEACOPP或ABVD）诱导治疗后仅获得PR，给予DLBCL样增强免疫化疗方案挽救治疗后均获得CR。3例患者接受增强的DLBCL样免疫化疗方案（R-CHOPE/EPOCH）诱导治疗，其中1例获得CR；1例获得PR后在二线治疗过程中进展，调整为BEACOPP挽救治疗后获得CR；1例诱导治疗中PD，行二线化疗联合CD30单抗Brentuximab Vedotin（BV）仍为难治状态，予苯达莫司汀联合PD1单抗挽救后终获得CR；3例患者达CR后均序贯自体造血干细胞移植（ASCT）巩固治疗。随访至今，1例患者失访，1例患者在诊断后36.9个月因疾病进展死亡，其余3例患者均维持持续缓解状态（表1）。

## 讨论及文献复习

GZL的中位发病年龄为30～40岁，男性多于女性，多表现为侵袭性病程[1],[4]–[5]。MGZL患者更多见，占GZL的43％～81％，临床表现为纵隔肿块，可伴有呼吸困难或上腔静脉阻塞综合征。而无纵隔受累的GZL（NMGZL）患者起病年龄多大于50岁，结外受累包括骨髓受累更多见[6]–[7]。我中心的5例患者临床特点与既往报道基本相符，性别分布可能与病例数有关。

GZL诊断困难，主要依赖病理形态和免疫表型。GZL具有宽泛的形态学谱系。在形态类似PMBCL的病例中，肿瘤细胞可大片状分布；而形态类似cHL的病例中，肿瘤细胞散在分布，背景散在小淋巴细胞、组织细胞和嗜酸性粒细胞，可伴有不同程度的纤维化和坏死灶，但中性粒细胞少见[1],[7]–[8]。免疫表型与形态学的不一致性是诊断的重要线索，形态学类似cHL的病例可呈现CD20强阳性、CD30阳性而CD15阴性；而形态学类似DLBCL的病例表现为CD30、CD15强阳性，CD20、CD79a缺失[7]–[8]。为了尽量减少误诊，初诊时应选取受累淋巴结或肿物切除活检而非穿刺，同时尽可能完善相关免疫组化标志和分子生物学检查以精确诊断。

在分子生物学特征方面，既往研究表明PMBCL和cHL均存在JAK-STAT和NF-κB信号通路激活及PD1/9p24.1和JAK2/PDL2扩增[9]–[10]。而Eberle等[11]对33例GZL进行遗传学分析也发现类似的免疫逃避位点基因异常，如33％和55％的患者分别具有REL/BCL11A和JAK2/PDL2位点扩增，27％的患者具有CIITA位点重排。DNA甲基化研究表明MGZL具有明显的表观遗传学特征，并且与结节硬化型cHL和PMBCL更相似[12]。近期Sarkozy等[13]对50例GZL样本进行了全外显子测序和靶向测序，结果显示GZL内部仍存在异质性，MGZL具有与cHL和PMBCL相似的突变特征，常见突变基因为SOCS1（45％）、B2M（45％）、TNFAIP3（35％）、GNA13（35％）、LRRN3（32％）和NFKBIA（29％）；而NMGZL主要富含与凋亡信号途径相关的突变，如TP53（39％）、BCL2（28％）、BIRC6（22％）等。这几项研究从不同角度说明MGZL与cHL/PMBCL存在相似的分子生物学特征，提示可能具有共同细胞起源。

在治疗方面，目前尚无GZL一线标准治疗方案。既往各医学中心一线治疗多选择治疗cHL的方案如ABVD或治疗DLBCL的方案如R-CHOP，几项回顾性研究显示一线ABVD治疗的CR率仅为30％～40％，而R-CHOP的CR率可达50％～71％[4],[6],[8]。一项多中心回顾性研究纳入112例GZL患者[6]，一线接受ABVD±R治疗的患者2年无事件生存（EFS）率明显低于CHOP±R/DA-EPOCH-R治疗的患者（22％对52％，*P*＝0.03），表明DLBCL样诱导方案对于初诊GZL患者可能是更好的选择。考虑到化疗强度可能影响疗效，首个前瞻性研究应用DA-EPOCH-R治疗24例初诊MGZL患者[14]，总体缓解（OR）率和CR率分别高达100％和79％，中位随访59个月，EFS率和OS率分别为62％和74％。Sarkozy等[8]报道的回顾性研究显示强化化疗组（大剂量R-CHOP/BEACOPP）较标准方案组（R-CHOP/ABVD）有更高的3年EFS率（74％对48％，*P*＝0.003）和OS率（90％对67％，*P*＝0.020），提示增强剂量的免疫化疗可能提高患者生存。本研究中3例GZL患者采取DLBCL样强化诱导治疗，2例取得PR以上疗效，因此，基于既往研究及本中心治疗经验，我们仍倾向于对年轻、体能状态良好的患者采取一线强化免疫化疗如DA-EPOCH-R。

GZL一线治疗后33％～58％的患者复发，且大部分发生在1年以内[6],[8],[14]。Wilson等[14]的研究中，9例患者早期复发，接受了受累野放疗（IFRT）后，4例患者获得持续缓解，表明对于仅纵隔复发的患者，IFRT可能是有效的挽救治疗手段之一。作为DLBCL的标准挽救治疗，大剂量化疗（HDT）联合ASCT在GZL中的作用有待验证，在一项回顾性研究中[15]，24例复发难治（R/R）患者在接受HDT联合ASCT治疗后获得65％的PFS率和75％的OS率。Evens等[6]分析了65例复发患者的挽救治疗疗效，其中61％的患者接受了造血干细胞移植（38％为异基因造血干细胞移植，62％为ASCT），接受移植组2年OS率明显优于未移植组（88％对67％，*P*＝0.010）。本研究中4例R/R患者接受挽救治疗后，3例患者获得CR并行ASCT巩固治疗，其中2例获得持续缓解。说明经过有效的挽救治疗，部分患者仍可再次获得缓解，而序贯造血干细胞移植巩固治疗可能降低患者的复发风险。

随着GZL生物学研究的深入，CD30和PD-1成为潜在的治疗靶点。一项Ⅱ期临床研究评估了CD30单抗BV单药治疗R/R B细胞非霍奇金淋巴瘤的疗效，其中6例GZL，1例获得了CR，2例获得了PR[16]。另一项研究使用BV联合R-CHP方案治疗初诊CD30阳性B细胞淋巴瘤，其中2例GZL患者均获得CR[17]。Melani等[18]应用PD-1抑制剂治疗3例R/R GZL患者，均获得CR。本研究中1例原发难治GZL患者接受三线苯达莫司汀联合PD-1单抗治疗后也获得CR。因此，CD30单抗和PD-1抑制剂有望为GZL治疗提供新的选择。

由于MGZL和NMGZL具有一定的临床及生物学差异，2022年WHO最新分类已建议将NMGZL划入DLBCL非特指型的范畴[2]，但考虑到GZL罕见，仍需积累更多的病例以认识两者之间的区别。综上所述，GZL发病率较低，诊断具有一定困难，诊断时应尽可能完善相关免疫组化标志和免疫表型。一线DLBCL强化免疫治疗方案可能疗效更好，R/R患者可考虑非交叉耐药的联合化疗或联合新药如BV或PD-1单抗，但未来需要更大样本的研究提供循证医学依据。
